# Duodenal Chromogranin A Cell Density as a Biomarker for the Diagnosis of Irritable Bowel Syndrome

**DOI:** 10.1155/2014/462856

**Published:** 2014-06-16

**Authors:** Magdy El-Salhy, Odd Helge Gilja, Doris Gundersen, Jan Gunnar Hatlebakk, Trygve Hausken

**Affiliations:** ^1^Section for Gastroenterology, Department of Medicine, Stord Hospital, P.O. Box 4000, 54 09 Stord, Norway; ^2^Section for Gastroenterology, Department of Clinical Medicine, University of Bergen, 5021 Bergen, Norway; ^3^National Centre for Ultrasound in Gastroenterology, Department of Medicine, Haukeland University Hospital, 5021 Bergen, Norway; ^4^Department of Research, Helse-Fonna, 5528 Haugesund, Norway

## Abstract

*Background and Aim*. Chromogranin A (CgA) is a common marker for endocrine cells. The density of duodenal CgA cells is reduced in patients with irritable bowel syndrome (IBS). *Methods*. The present study was undertaken to evaluate the density of duodenal CgA as a biomarker for the diagnosis of IBS. Two hundred and three patients with IBS were recruited (180 females and 23 males; mean age, 36 years; range, 18–66 years). The control group comprised 86 healthy subjects without gastrointestinal complaints (77 females and 9 males; mean age, 38 years; range, 18–67 years). Biopsy samples were taken from the duodenum during gastroscopy. Sections from these biopsy samples were immunostained for CgA using the avidin-biotin complex (ABC) method. CgA cell density was quantified by computerized image analysis. *Results*. The CgA cell density was lower in IBS-total and in all of the IBS subgroups than in the controls. The sensitivity and specificity for a cutoff of <200 cells/mm^2^ were 86% and 95%, respectively. *Conclusion*. The duodenal CgA cell density seems to be a good biomarker for the diagnosis of IBS. It is an inexpensive, simple, and easy-to-use method that does not require sophisticated equipment or considerable experience.

## 1. Introduction

Irritable bowel syndrome (IBS) is a common gastrointestinal disorder that is characterized by recurrent abdominal pain/discomfort and altered bowel habits (ranging from diarrhea to constipation) and abdominal bloating [1]. The results of physical examinations, blood tests, and radiological and endoscopic examinations are normal in IBS patients. The diagnosis of IBS in clinical practice has therefore been a diagnosis of exclusion, where an extensive battery of examinations and tests is conducted to exclude organic diseases that could be responsible for the patient's symptoms [2–5]. Since 1978, several attempts have been made to achieve a positive diagnosis based on symptom assessments similar to those used in psychiatry [6–13]. In 1988, an international panel of experts introduced symptom-based criteria for the diagnosis of IBS, known as Rome I criteria, which were succeeded by refinements in 1999 (Rome II criteria) and 2006 (Rome III criteria). According to Rome III criteria, IBS patients are divided into three subtypes based on their stool pattern: patients with diarrhea as the predominant symptom (IBS-D), with both diarrhea and constipation (IBS-M), and with constipation as the predominant symptom (IBS-C).

The aims of Rome diagnostic criteria were to achieve a positive diagnosis, avoid unnecessary tests and examinations, and optimize treatment. Although more than 2 decades have passed since the introduction of the Rome criteria, they are not widely used in everyday clinical practice [3, 14, 15]. The main reason that the Rome criteria have failed to achieve their goals is the potential to miss organic diseases that mimic IBS symptoms, but which have different pathophysiology and treatments. Furthermore, they are not applicable in real, everyday clinical practice [2, 3, 14, 15]. There is a consensus among gastroenterologists that a diagnostic biomarker for IBS is urgently needed [16–18].

The duodenum harbors a large number of endocrine cells and comprises numerous endocrine cell types [19]. It has been reported that the populations of several duodenal endocrine cell types are reduced in patients with IBS [20]. Chromogranin A (CgA) is a common marker for these endocrine cells [21–23], and consequently it has been found that CgA is reduced in these patients [24]. It has been suggested that the duodenal CgA cell density could be used in the diagnosis of IBS patients [24], with a sensitivity and specificity of 91% and 89%, respectively [5]. However, these results were based on 41 IBS patients that included only IBS-D and IBS-C subtypes and 42 controls. Thus, the present study was undertaken to test the effectiveness of duodenal CgA cell density as a marker for the diagnosis of IBS using a large cohort of IBS patients including all IBS subtypes and a large number of healthy subjects as controls.

## 2. Material and Methods

### 2.1. Patients and Controls

Two hundred and three patients who fulfilled Rome III criteria for the diagnosis of IBS [6, 7] were recruited from patients referred to Stord Hospital during 2002–2011. These patients comprised 180 females and 23 males with a mean age of 36 years (range, 18–66 years). The subtypes of IBS in these patients were distributed as follows: 80 with IBS-D, 47 with IBS-M, and 76 with IBS-C. All of the patients had a long duration of IBS symptoms and a symptom onset that was not associated with any gastrointestinal or other infections. All patients underwent a complete physical examination and were investigated using the following blood tests: full blood count, electrolytes, inflammatory markers, liver tests, and thyroid function tests. They also underwent further colonoscopy with segmental biopsies to exclude microscopic colitis.

The control group comprised 86 healthy subjects without gastrointestinal complaints (77 females and 9 males; mean age, 38 years; range, 18–67 years). Of these subjects, 59 were healthy volunteers who had no gastrointestinal complaints and were recruited via local announcements at Stord Hospital, Haukelands University Hospital, and the University of Bergen, as well as in local newspapers. Fifteen were from the population of Stord city and 44 were students or hospital employees. Twenty-seven healthy subjects submitted to gastroscopy because of health worries due to a relative being diagnosed with cancer.

The study was performed in accordance with the Declaration of Helsinki and was approved by the Regional Committee for Medical and Health Research Ethics West, Bergen, Norway. All subjects provided oral and written consent to participate.

### 2.2. Gastroscopy, Histopathology, and Immunohistochemistry

Gastroscopy was performed on both the patients and the controls after an overnight fast. During gastroscopy, four biopsy samples were taken from the descending part of the duodenum, distal to the papilla of Vateri. Two additional biopsy samples were taken from the antrum and used for a rapid urease test to identify the presence of* Helicobacter pylori *(HelicotecUT Plus, Strong Biotech, Taipei, Taiwan).

The biopsy samples were fixed overnight in 4% buffered paraformaldehyde, embedded in paraffin, and sectioned at a thickness of 5 *μ*m. The sections were stained with hematoxylin-eosin and immunostained using the avidin-biotin complex (ABC) with the VECTASTAIN ABC kit (Vector Laboratories, Burlingame, CA, USA). The primary antibody used was a monoclonal mouse antibody raised against the N-terminal of purified CgA (code no. M869, Dako, Glostrup, Denmark). The sections were hydrated and then immersed in 0.01% hydrogen peroxide in PBS buffer (pH 7.4) for 10 min to inhibit endogenous peroxidase activity. After washing in buffer, the sections were treated with 1% bovine serum albumin for 30 min to block nonspecific binding sites and then incubated with the primary antibody diluted to 1 : 500 at room temperature for 1 h. The sections were washed in PBS buffer and incubated with biotinylated swine anti-mouse IgGdiluted 1 : 200 for 30 min at room temperature. After washing the slides in PBS buffer, the sections were incubated for 30 min with avidin-biotin-peroxidase complex diluted to 1 : 100 and then immersed in 3,3′-diaminobenzidine peroxidase substrate (Vector laboratories), followed by counterstaining in hematoxylin [8].

### 2.3. Quantification of CgA Cells

The CgA cell density was quantified by computerized image analysis and by counting positive cells in a microscopic field. Measurements using computerized image analysis were performed on a computer linked to a microscope (BX 43, Olympus) equipped with a digital camera (DP 26, Olympus). The number of immunoreactive cells and the area of the epithelial cells were measured. The number of endocrine cells in each field was counted manually by pointing and clicking the computer mouse, and the area of the epithelium containing these cells was drawn manually using the computer mouse. A ×40 objective was used, for which each frame (field) on the monitor represented a tissue area of 0.14 mm^2^. CgA cells were measured in ten randomly chosen fields.

CgA-positive cells were counted in ten and five randomly chosen microscopic fields using a ×40 objective. Immunostained sections from the IBS patients and controls were coded and mixed, and measurements were made by the same person (M.E.), who was blind to the identity of the sections. The data from the fields were tabulated, and the cell density of the epithelium (in cells per square millimeter) and number of cells per microscopic field were computed.

### 2.4. Statistical Analysis

Differences in gender and the incidence of* H. pylori* infection between the patients and controls were tested by chi-square and Fisher's exact tests, respectively. Differences in the age profile were tested by the Mann-Whitney nonparametric test. Differences between controls, all IBS patients (IBS-total), IBS-D, IBS-M, and IBS-C patients were tested using the Kruskal-Wallis nonparametric test with Dunn's posttest. The data are presented as mean ± SEM values, and differences with *P* < 0.05 were considered to be statistically significant.

## 3. Results

### 3.1. Patients and Controls

The gender and age distributions did not differ significantly between the patients and the controls (*P* = 1.0 and *P* = 0.6, resp.).* H. pylori* infection was found in 12 of the patients and 8 of the control subjects (as evidenced by both the urease test and by histopathological examination), and its incidence did not differ between the two groups (*P* = 0.6).

### 3.2. Gastroscopy, Histopathology, and Immunohistochemistry

The endoscopic findings were normal in both the patients and the controls, and histopathological examination of the duodenum revealed normal histology in all cases. CgA-immunoreactive cells were found mostly in the crypts of the duodenum of both the patients and the controls and were basket- or flask-shaped, sometimes with a long basal cytoplasmic process.

### 3.3. Quantification of CgA Cells

**Figure 1:**
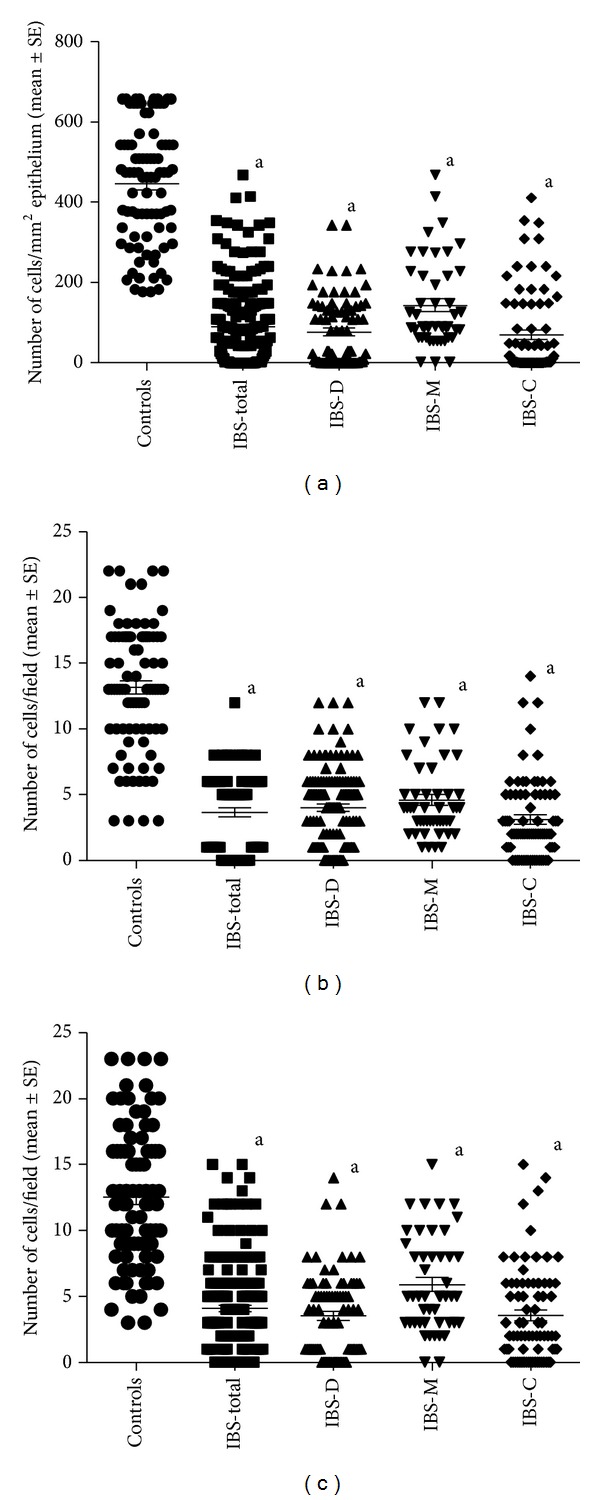
CgA cell density expressed in relation to epithelial cells, measured (a) in ten randomly chosen fields, (b) as the number of cells per microscopic field in ten randomly chosen fields, and (c) as the number of cells per microscopic field in five randomly chosen fields in tissue samples taken from controls and IBS-total, IBS-D, IBS-M, and IBS-C patients. ^a^ *P* < 0.0001* versus* control group.

**Figure 2:**
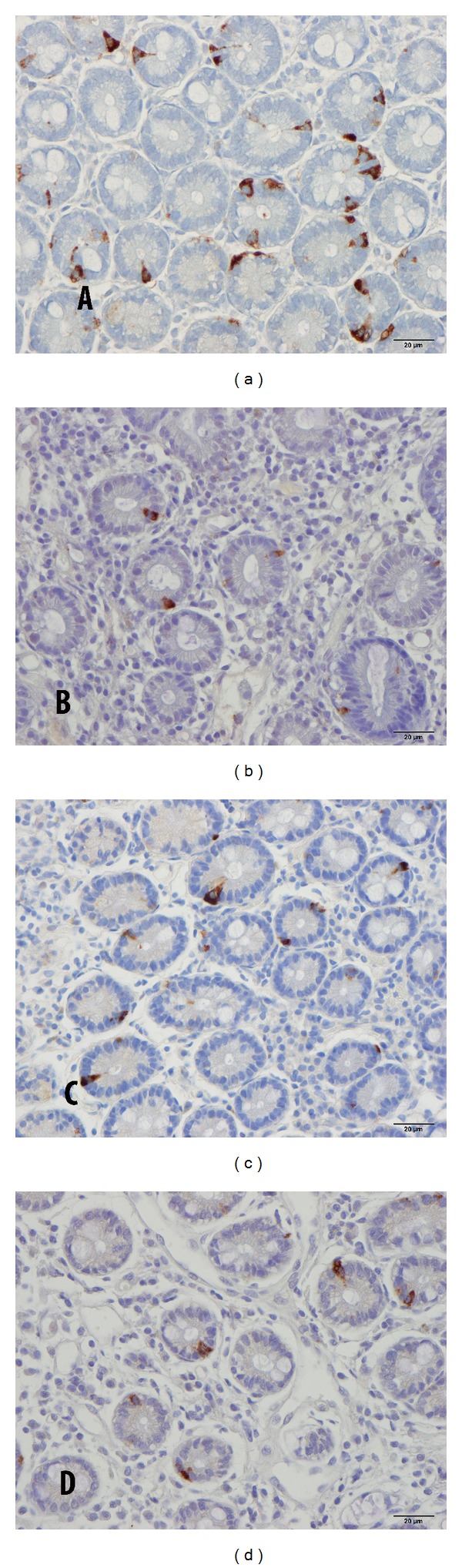
Duodenal CgA-immunoreactive cells in (a) a control subject, (b) a patient with IBS-D, (c) a patient with IBS-M, and (d) a patient with IBS-C.

**Figure 3:**
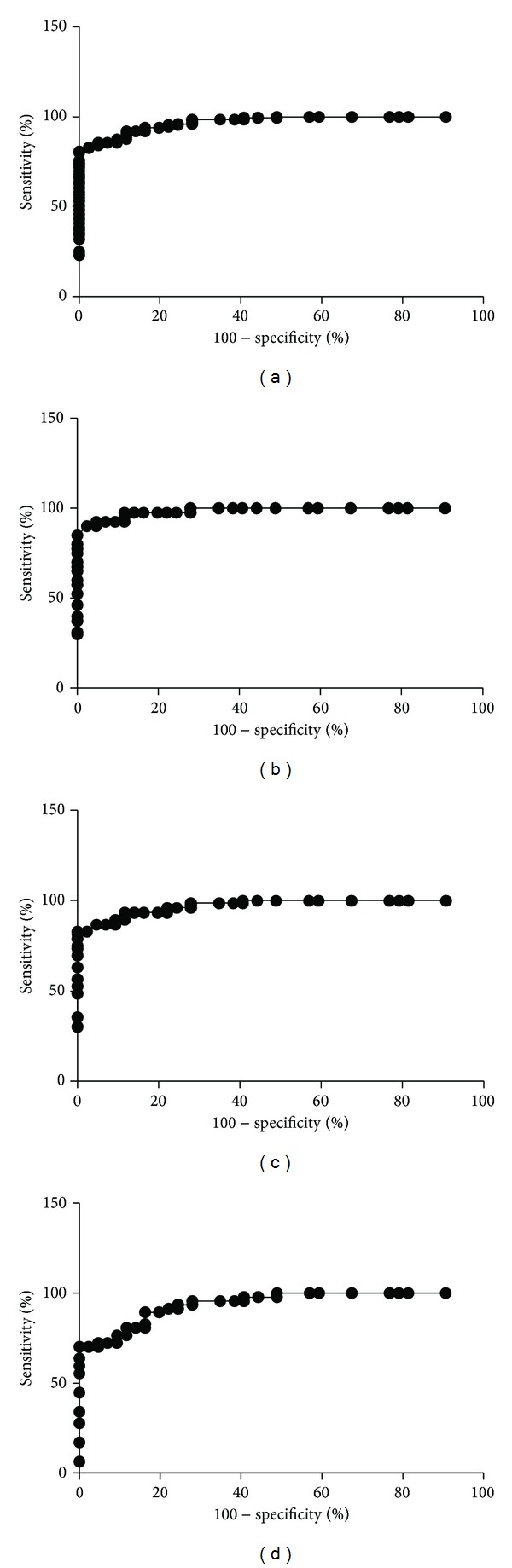
ROC analysis for CgA cell density as measured relative to the area of the epithelium in (a) IBS-total, (b) IBS-D, (c) IBS-M, and (d) IBS-C.

Computerized image analysis yielded CgA cell densities of 446.1 ± 16.0, 89.5 ± 7.2, 76.7 ± 9.6, 142.9 ± 15.9, and 69.8 ± 100.5 cells/mm^2^ for controls and IBS-total, IBS-D, IBS-M, and IBS-C patients, respectively (Figures 1 and 2). The Kruskal-Wallis test was significant (*P* < 0.001). Dunn's posttest revealed that CgA cell density was lower in IBS-total and all of the IBS-subgroups relative to the controls (*P* < 0.0001 for all). Receiver operator curve (ROC) analysis for CgA cell density in the duodenum revealed that the sensitivity and specificity for a cutoff of <200 cells/mm^2^ were 86% and 95%, respectively, in IBS-total, 93% and 95% in IBS-D, 72% and 95% in IBS-M, and 87% and 95% in IBS-C (Figure 3).

**Figure 4:**
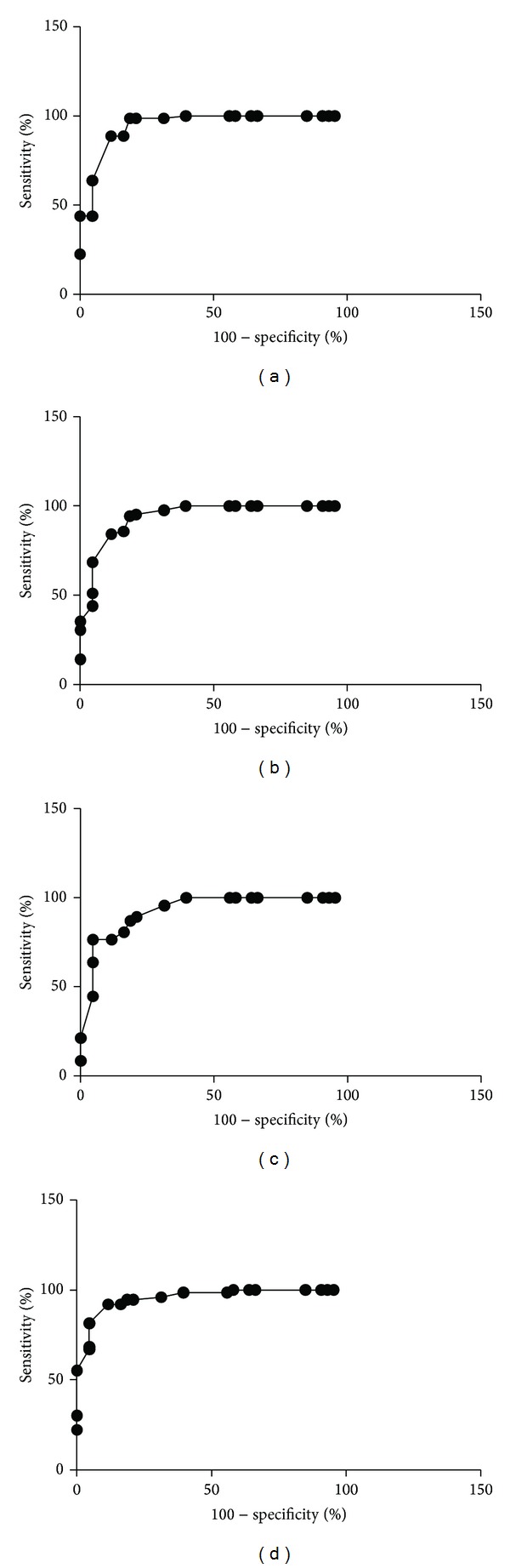
ROC analysis for CgA cell density as measured in ten randomly chosen microscopic fields in (a) IBS-total, (b) IBS-D, (c) IBS-M, and (d) IBS-C.

The numbers of CgA cells in ten microscopic fields were 13 ± 1.0, 4 ± 0.3, 4 ± 0.3, 5 ± 0.4, and 3 ± 0.3 cells/field for controls and IBS-total, IBS-D, IBS-M, and IBS-C patients, respectively (Figures 1 and 2). The Kruskal-Wallis test revealed a statistically significant difference between the controls and the IBS-total, IBS-D, IBS-M, and IBS-C patients (*P* < 0.0001 for all). According to Dunn's multiple comparison test, the number of CgA cells was significantly lower in the IBS-total, IBS-D, IBS-M, and IBS-C patients than in the controls (*P* < 0.0001 for all). ROC analysis showed that the sensitivity and specificity for a cutoff of <6 cells/field were 89% and 88%, respectively, in IBS-total, 84% and 88% in IBS-D, 77% and 88% in IBS-M, and 92% and 88% in IBS-C (Figure 4).

**Figure 5:**
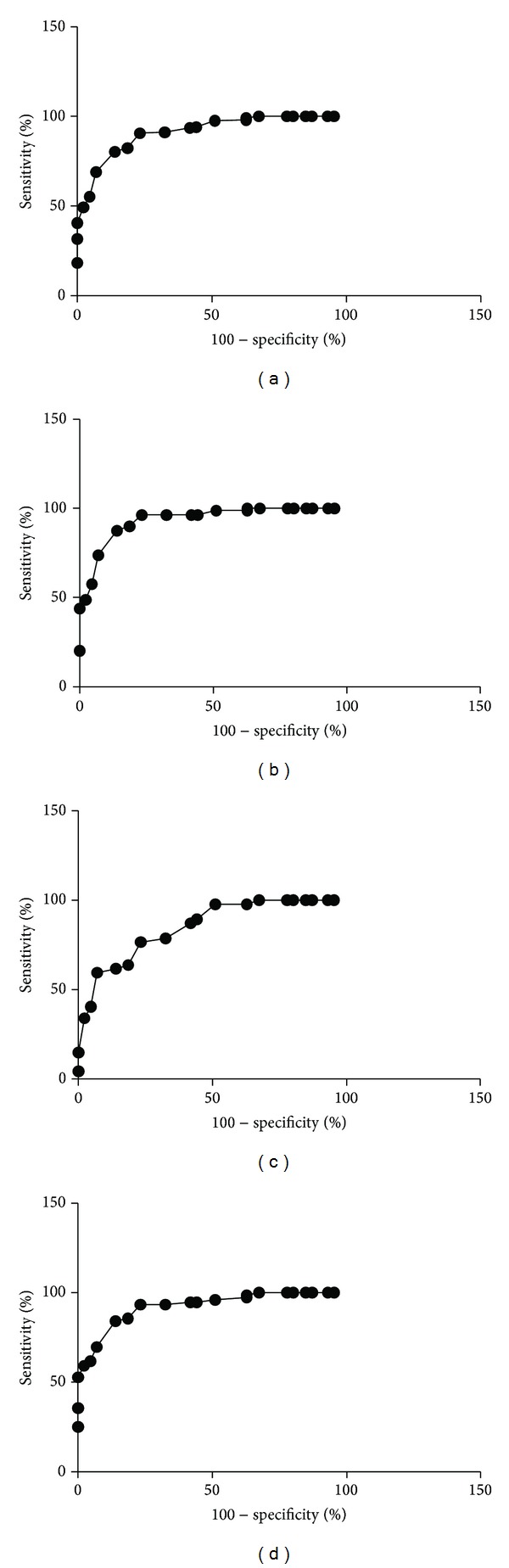
ROC analysis for CgA cell density as measured in five randomly chosen microscopic fields in (a) IBS-total, (b) IBS-D, (c) IBS-M, and (d) IBS-C.

The numbers of CgA cells in five microscopic fields were 13 ± 1.0, 4 ± 0.3, 4 ± 0.4, 6 ± 0.5, and 4 ± 0.4 cells/field for controls and IBS-total, IBS-D, IBS-M, and IBS-C patients, respectively (Figures 1 and 2). The Kruskal-Wallis test revealed a statistically significant difference between the controls and the IBS-total, IBS-D, IBS-M, and IBS-C patients (*P* < 0.001 for all). Dunn's test showed that the number of CgA cells was significantly lower in the IBS-total, IBS-D, IBS-M, and IBS-C patients than in the controls (*P* < 0.0001 for all). ROC analysis showed that the sensitivity and specificity for a cutoff of <6 cells/field were 80% and 86%, respectively, in IBS-total, 88% and 86% in IBS-D, 62% and 86% in IBS-M, and 88% and 86% in IBS-C (Figure 5).

## 4. Discussion

A wide range of biomarkers that reflect a pathological state in IBS has been considered for the diagnosis of IBS [15–18]. This has prompted evaluations of several tests and examinations measuring gut motility, visceral hypersensitivity, autonomic reactivity, mucosal inflammation, fecal proteases, gut flora, serum antibodies, and food allergy [15–18]. Unfortunately, none of these tests or examinations has been found to be useful as a biomarker for IBS diagnosis.

The number of endocrine cells in the gastrointestinal tract is the largest in the duodenum, followed by the rectum [25]. Moreover, the duodenum contains the largest number of gut endocrine cell types, namely, serotonin, secretin, cholecystokinin (CCK), gastric inhibitory polypeptide (GIP), somatostatin, and motilin [20]. Of these, the densities of four cell types were shown to be reduced in patients with IBS: secretin, CCK, GIP, and somatostatin cells [8]. The reduction in the densities of these cells may account for the reported reduction in the density of duodenal CgA cells in IBS patients [9]. The duodenal CgA cell density thus reflects structural abnormality occurring in the duodenal endocrine cells of IBS patients [26].

The present study showed that duodenal CgA cell density is a biomarker with good sensitivity and specificity for the diagnosis of IBS. The use of computerized image analysis provided slightly improved sensitivity and specificity than direct counting of cell numbers in microscopic fields. However, computerized image analysis requires equipment that might not be available in small pathological laboratories. Considering the small gain in sensitivity and specificity, direct counting with the aid of a microscope is preferable, especially in small laboratories. As counting of ten randomly chosen fields has better sensitivity and specificity than counting in five fields, the former should be used. The sensitivity of this biomarker seems to be lower for the IBS-M subtype than for the other IBS-C and IBS-D subtypes. The densities of duodenal endocrine cells in patients with celiac disease have been reported to be increased [10]. The densities of the duodenal endocrine cells in other diseases/disorders such as duodenitis, gastritis, inflammatory bowel diseases, duodenal and stomach ulcers, and gastrointestinal malignancy are not known. Thus, the present observations do not exclude the possibility that the alteration in CgA cell density may occur in such diseases/disorders. Further studies on these patients' group are needed before reaching any definite conclusion.

Screening of IBS patients for celiac disease (CD) is now widely accepted [27–37]. Thus, gastroscopy with duodenal biopsies can be used instead of serology for excluding or confirming a CD diagnosis, and the same biopsies can be used for the diagnosis of IBS. Gastroscopy is generally accepted by patients and immunohistochemistry is a routine method in all pathological laboratories. Manually counting CgA cells in ten microscopic fields is not time consuming and does not require sophisticated equipment or considerable experience. Immunohistochemical staining for CgA is not expensive and is performed commonly in pathology laboratories for the diagnosis of endocrine tumors.
